# Tracking and clarifying differential traits of classical- and atypical L-type bovine spongiform encephalopathy prions after transmission from cattle to cynomolgus monkeys

**DOI:** 10.1371/journal.pone.0216807

**Published:** 2019-05-16

**Authors:** Ken’ichi Hagiwara, Yuko Sato, Yoshio Yamakawa, Hideyuki Hara, Minoru Tobiume, Yuko Okemoto-Nakamura, Tetsutaro Sata, Motohiro Horiuchi, Hiroaki Shibata, Fumiko Ono

**Affiliations:** 1 Department of Biochemistry and Cell Biology, National Institute of Infectious Diseases, Shinjuku-ku, Tokyo, Japan; 2 Department of Pathology, National Institute of Infectious Diseases, Shinjuku-ku, Tokyo, Japan; 3 Laboratory of Veterinary Hygiene, Faculty of Veterinary Medicine, Graduate School of Infectious Diseases, Hokkaido University, Sapporo, Hokkaido, Japan; 4 Tsukuba Primate Research Center, National Institutes of Biomedical Innovation, Health and Nutrition, Tsukuba, Ibaraki, Japan; University of Verona, ITALY

## Abstract

Classical- (C-) and atypical L-type bovine spongiform encephalopathy (BSE) prions cause different pathological phenotypes in cattle brains, and the disease-associated forms of each prion protein (PrPSc) has a dissimilar biochemical signature. Bovine C-BSE prions are the causative agent of variant Creutzfeldt-Jakob disease. To date, human infection with L-BSE prions has not been reported, but they can be transmitted experimentally from cows to cynomolgus monkeys (*Macaca fascicularis*), a non-human primate model. When transmitted to monkeys, C- and L-BSE prions induce different pathological phenotypes in the brain. However, when isolated from infected brains, the two prion proteins (PrPSc) have similar biochemical signatures (i.e., electrophoretic mobility, glycoforms, and resistance to proteinase K). Such similarities suggest the possibility that L-BSE prions alter their virulence to that of C-BSE prions during propagation in monkeys. To clarify this possibility, we conducted bioassays using inbred mice. C-BSE prions with or without propagation in monkeys were pathogenic to mice, and exhibited comparable incubation periods in secondary passage in mice. By contrast, L-BSE prions, either with or without propagation in monkeys, did not cause the disease in mice, indicating that the pathogenicity of L-BSE prions does not converge towards a C-BSE prion type in this primate model. These results suggest that, although C- and L-BSE prions propagated in cynomolgus monkeys exhibit similar biochemical PrPSc signatures and consist of the monkey amino acid sequence, the two prions maintain strain-specific conformations of PrPSc in which they encipher and retain unique pathogenic traits.

## Introduction

Transmissible spongiform encephalopathies (TSEs) are fatal neurodegenerative disorders that affect humans and several other mammalian species. The causative agents are referred to as ‘prions’, which principally consist of disease-associated forms of prion protein (PrPSc; PrP denotes prion protein) [1]. PrPSc is a pathogenic conformational isoform of otherwise non-pathogenic cellular prion protein (PrPC). PrPC is an *N*-glycosylated and glycosylphosphatidylinositol-anchored protein encoded by the host gene *Prnp* [2]. Although PrPC is digested by proteinase K (PK), PrPSc is partially resistant to digestion, and its carboxyl-terminus remains undegraded. Conversion of PrPC to PrPSc is seeded by pre-existing PrPSc. The seeds are acquired initially by unknown processes acting on endogenous PrPC, by mutation of *Prnp*, or by intake of external PrPSc. Accordingly, TSEs emerge as sporadic, hereditary, and infectious diseases.

Prion strains exist that exhibit pathogenic and biochemical variations, such as ranges of susceptible host species, incubation periods, lesion profiles in the brain, and PrPSc glycoform profiles and electrophoretic mobility. It is presumed that strain diversity is due to distinct structures of PrPSc. Several structural models of PrPSc have been proposed, however, elucidation of PrPSc structures is challenging because of the insolubility and heterogeneity of PrPSc [3–5]. Of note, transmission of prions to new host animal species (referred to as ‘interspecies transmission’) occasionally results in strain ‘mutation’ via heritable conformational changes of parental strains ([6–8], and reviewed in [9]). Such mutation is thought to take place due to moderate conformational fluctuations of small seeding-aggregates of PrPSc that develop and adapt to the spectra of conformations of PrPC of the new host animal species [9].

To date, three different types of bovine spongiform encephalopathy (BSE) have been identified: classical-BSE (C-BSE), and two sparse atypical types caused by L- [10] and H-BSE prions [11]. Although C-BSE prions in cattle are known to cause variant Creutzfeldt-Jakob disease (vCJD) [12–15], human infection with atypical BSE prions has not been reported. However, as L-BSE prions are transmissible to non-human primates [16–18] and to transgenic mice expressing a human form of PrPC [19–22], L-BSE prions may have zoonotic potential.

In previous studies, infection of cynomolgus monkeys with C-BSE prions via intracranial inoculation caused deposition of florid plaques of PrPSc in the brain. These plaques are also observed in the brains of vCJD patients [13, 23]. Conversely, cynomolgus monkeys infected with L-BSE prions were devoid of florid plaques of PrPSc in the brain [16, 17]. In spite of the different pathological phenotypes caused by inoculation of C- and L-BSE prions, both types of accumulated PrPSc in the brains of monkeys showed similar electrophoretic mobility and glycoform profiles. Such biochemical similarities raised a question regarding the identity of L-BSE prions after transmission to monkeys. Indeed, several studies have found that L-BSE prions transform into a C-BSE prion type, or C-BSE prion-like agents following interspecies transmission from cows [19, 24–26]. In addition, a previous study in cynomolgus monkeys discussed the necessity of examining the stability and pathogenicity of L-BSE prions upon transmission from cows to a primate model [16]. Therefore, we examined the traits of C- and L-BSE prions isolated from cows or cynomolgus monkeys, to explore whether propagation of L-BSE prions in cynomolgus monkeys leads to emergence of C-BSE prions.

## Materials and methods

### Ethics statement

Brain tissues of cynomolgus monkeys were obtained from our previous studies [17, 23], which were conducted at the Tsukuba Primate Research Center (TPRC) of the National Institutes of Biomedical Innovation, Health and Nutrition, Japan (NIBIOHN), according to TPRC of NIBIOHN rules for animal care and management, and the guiding principles for animal experiments using non-human primates formulated by the Primate Society of Japan [27]. Monkeys in these studies were from the breeding colony of TPRC, and protocols for prion transmission from cows to monkeys were approved by the animal welfare, animal care and use committee, and the animal ethics biosafety committee of NIBIOHN (approval numbers: DK17-003, DS18-069, and DS18-069R1). Monkeys were anesthetized with ketamine/xylazine, and intracranially inoculated with brain homogenates of BSE-affected cows. Monkeys were housed in individually ventilated cages of appropriate size. The cages were designed and placed to allow monkeys in neighboring cages to have good visual and auditory communication, and to view adjoining spaces. The room was maintained at 25 ± 2°C, with a relative humidity of 60 ± 10%, and a 12-h light-and-dark cycle (7 p.m. to 7 a.m. in the dark). Monkeys were fed 70 g of commercial animal chow (Type AS; Oriental Yeast Co., Ltd., Tokyo, Japan) and 100 g of apples daily. Water was supplied *ad libitum*. At humane endpoints to minimize distress, monkeys were sedated with ketamine and euthanized by intravenous administration of sodium pentobarbital. Tissue samples were then collected. Tissues from BSE-affected cows were obtained post mortem from local abattoirs and meat inspection centers where naturally occurring BSE cases were identified by post-mortem BSE screening. The use of cow brain samples as inocula was approved by the ethics committee for animal experiments of the National Institute of Infectious Diseases, Japan (NIID) (approval numbers: 106041, 207114, 208041, 209040, and 210067), and by the animal welfare and animal care and use committees, and the animal ethics biosafety committee of NIBIOHN (approval numbers: DK17-003, DS18-069, and DS18-069R1). All experiments in mice were carried out in compliance with the laws and guidelines of the Ministry of Environment, Japan, the guidelines of the Ministry of Education, Culture, Sports, Science and Technology, Japan, and the guidelines of NIID. Protocols for transmission of prions to mice were approved by the ethics committee for animal experiments of NIID (approval numbers: 106041, 207114, 208041, 209040, 210067, 111074, 212090, 213118, and 116070).

### Mice, inoculation, and observation

Female C57BL/6J mice and SJL/J mice were purchased from Charles River Laboratories Japan, Inc. (Kanagawa, Japan), and RIIIS/J mice were purchased from Jackson Laboratories (JAX stock number: 000683, Bar Harbor, Maine, USA). Mice between 6 and 10 weeks old were anesthetized with isoflurane and intracranially injected once near the bregma at a depth of 3 mm from the surface of the skull, with 25-μL aliquots of inocula prepared from bovine brains, monkey brains, or mouse brains as described below. Mice were cared for according to the biosafety regulations and the ethical statements of NIID, examined weekly for neurological signs until the presymptomatic phase, and observed daily or every other day thereafter. Experiments were terminated at humane endpoints to minimize distress, determined by criteria including body weight loss, trotting, ataxia of gait, or any neurological or non-neurological manifestations. Mice were euthanized by inhalation of isoflurane. The brain, spleen, and ileum were taken for Western blot analysis, and histopathological and immunohistochemical examinations.

### Inocula

#### Bovine brain

Homogenates of the thalamus of a C-BSE-affected Holstein cow (83 months old, case number: JP6, identical to case number 2 in [28]), and the cerebrum of an L-BSE-affected Japanese black cow (169 months old, case number: JP24 [29]) were used as inocula to transfer prions from cows to mice. These cows were identified by national surveillance for BSE. Bovine brain tissues were homogenized at a concentration of 10% (w/v) in phosphate-buffered saline (PBS) (SAFC Biosciences, St. Louis, MO, USA) by vigorous shaking with ceramic YTZ beads (1.5-mm diameter balls, Nikkato Co., Osaka, Japan) at 2,500 rpm for 5 min in a Multi-beads shocker tissue disruptor (Yasui Kikai Co., Osaka, Japan) to prepare stock bovine brain homogenates. Concentrations of PrPSc in stock homogenates were measured by Western blot analysis. Before injection into mice, the homogenates were diluted in saline (Otsuka Pharmaceutical Factory, Inc., Tokushima, Japan) to prepare inocula with equivalent concentrations of PrPSc (S1 Fig); the brain homogenate of the C-BSE cow was diluted to a tissue concentration of 0.3% (w/v) and the brain of the L-BSE cow was diluted to a tissue concentration of 1% (w/v). Saline was used as an inoculation control.

#### Monkey brain

The frontal cortex of the cerebrum from a monkey (ID number #007) inoculated with bovine C-BSE prions [23], and a monkey (ID number #015) inoculated with bovine L-BSE prions [17], were homogenized at a concentration of 20% (w/v) in PBS using the ceramic beads described above. The brain homogenate of monkey #007 was diluted to a tissue concentration of 0.4% (w/v) and that of monkey #015 was diluted to a tissue concentration of 1% (w/v), such that the inocula contained equivalent concentrations of PrPSc for inoculation of mice according to Western blot analyses (S1 Fig). A whole brain homogenate from a healthy monkey (1% tissue in saline, w/v) was prepared for use as a control.

#### Mouse brain

Whole mouse brains were homogenized in PBS at a concentration of 20% (w/v) using the ceramic beads described above. The homogenates were further diluted in saline to a tissue concentration of 1% (w/v) for inoculation. To compare pathogenicities of bovine C-BSE prions and monkey C-BSE prions after their first passage in C57BL/6 mice, concentrations of PrPSc in the inocula were adjusted based on Western blot analysis (S1 Fig). The brain homogenate of the mouse infected by bovine C-BSE prions was diluted to a tissue concentration of 1% (w/v) in saline, and that of the mouse infected by monkey L-BSE prions was diluted to a tissue concentration of 0.8% (w/v) and supplemented with the brain homogenate of a healthy mouse to raise the total tissue concentration to 1% (w/v) in saline. A brain homogenate from a healthy mouse (1% tissue in saline, w/v) was prepared for use as a control.

### *Prnp* sequences

The *Prnp* coding regions of the cows used in this study had codons at glutamine78 and asparagine192 that were synonymous with those found in the *Bos taurus Prnp* sequence in a public database (accession number: AJ298878), as previously described [29]. To examine the *Prnp* sequence(s) of cynomolgus monkeys, DNA fragments containing *Prnp* coding regions were amplified from genomic DNA by PCR using KOD-plus DNA polymerase (Toyobo Co., Ltd., Osaka, Japan) with the following primers; 5’-TTC ATC AAG TCC ATA ACT TAG GGT CAG-3’ (forward primer) and 5’-CCT ATC AGG GAC AAA GAG AGA AGC AAG-3’ (reverse primer). DNA sequence analysis was carried out following the BigDye cycle sequencing protocol (Applied Biosystems Inc., California, USA). By comparison with the publicly curated *Prnp* sequence of *Macaca fascicularis* (Ensembl accession number: ENSMFAG00000033814), heterozygotic synonymous codons were found at tyrosine145 (TAT/TAC) and tyrosine163 (TAT/TAC) in monkey #007, and heterozygotic synonymous codons of proline39 (CCA/CCG), cysteine179 (TGT/TGC), and threonine192 (ACT/ACC) in monkey #015. The *Prnp* coding region of a negative control monkey (a monkey not inoculated with prions) was identical to the publicly curated sequence. DNA fragments containing the *Prnp* coding region of mice were obtained similarly using the following primers; 5’-ATG ACT TTC ATA CAT TTG CTT TGT AGA TAG-3’ (forward primer) and 5’-CCA GCA GTT ATT TGG TGT TAT ATT CTT ATT-3’. DNA sequence analysis confirmed that C57BL/6J, SJL/J, and RIIIS/J mice had the *Prnpa* genotype, as reported previously [30]. Detailed analysis confirmed that the coding region of *Prnp* of SJL/J mice was identical to the publicly curated sequence from *Mus musculus* C57BL/6J (accession number: OTTMUSG00000014846), and RIIIS/J mice had the homozygous synonymous codon GTT for valine188 instead of the homozygous codon GTC found in C57BL/6J and SJL/J mice.

### Digestion by PK

Mouse brains were homogenized at a concentration of 20% (w/v) in 50 mM HEPES-NaOH buffer containing 100 mM NaCl (pH 7.4) by vigorous shaking with ceramic beads (1.5 mm in diameter) at 2,500 rpm for 3 min in the Multi-beads shocker tissue disruptor. Thirty microliter aliquots of the homogenates were mixed with 30 μL of a buffer consisting of 4% (w/v) zwittergent 3–14 (Merck Millipore, Darmstadt, Germany), 1% (w/v) lauroylsarcosine sodium salt (Sigma-Aldrich, St. Louis, MO, USA), 1 mg/mL of collagenase (Wako Pure Chemical Industries, Osaka, Japan), 0.1 unit/μL of DNase I (Roche Diagnostics, Basel, Switzerland), 100 mM NaCl, and 50 mM HEPES-NaOH (pH 7.4). Samples were incubated at 37°C for 30 min. Samples were then mixed with PK (recombinant, Roche Diagnostics) to a final concentration of 50 μg/mL of PK, and incubated at 37°C for 30 min. After addition of 30 μL of a mixture of 2-butanol and methanol (5/1, v/v) containing 10 mM phenylmethanesulfonyl fluoride (Sigma-Aldrich) to stop the digestion, digests were centrifuged at 18,000 x g for 10 min at 23°C. Pellets were air dried and stored at -75°C until analysis. For PK digestion of mouse spleens and distal regions of the ileum, tissue homogenates were prepared at a concentration of 20% (w/v) by shaking with ceramic beads (2.7 mm in diameter) at 2,500 rpm for 5 min. Thirty microliter aliquots of the homogenates were treated with collagenase and DNase I as described above, then digested with PK at a final concentration of 50 μg/mL at 37°C for 30 min. Digests were mixed with 30 μL of 2-butanol/methanol (5/1, v/v) and centrifuged at 18,000 x g for 15 min at 23°C. Pellets were resuspended in 60 μL of a buffer consisting of 2% (w/v) zwittergent 3–14, 0.5% (w/v) lauroylsarcosine, 100 mM NaCl, and 50 mM HEPES-NaOH (pH 7.4), and further digested with PK at a final concentration of 5 μg/mL at 37°C for 30 min. Samples were mixed with phenylmethanesulfonyl fluoride, and precipitated by centrifugation, as described above.

Digestion of homogenates from cattle and monkey brains by PK was carried out in a similar manner as that for the mouse brains. In the analysis of PrPSc resistance to varying concentrations of PK, treatment with collagenase and DNase I was omitted.

### Western blot analysis

Pellets of PK-digested tissues were dissolved in lithium dodecyl sulfate sample buffer (Thermo Fisher Scientific Inc., Novex, Waltham, MA, USA) supplemented with 80 mM dithiothreitol, and heated at 100°C for 5 min to denature proteins. If removal of *N*-glycans from PrPSc was needed, heat-denatured samples were further incubated with peptide-*N*-glycosidase F (PNGase F, New England BioLabs, Ipswich, MA, USA) according to the manufacturer’s protocol. Aliquots of PK-digested samples before or after PNGase F treatment were subjected to sodium dodecyl sulfate-polyacrylamide gel electrophoresis (SDS-PAGE) using NuPAGE Novex Bis-Tris gels in a morpholinoethanesulfonic acid (MES)-NaOH running buffer (Thermo Fisher Scientific Inc., Invitrogen). After electrophoresis, proteins were transferred to Invitrolon PVDF membranes (Invitrogen) at 30 V for 90 min using NuPAGE transfer buffer (Invitrogen) supplemented with 10% (v/v) methanol and 0.01% (v/v) SDS. SDS-PAGE was also carried out using a modified Tris-HCl/Tris-glycine buffer system [31] in which the concentrations of gel buffer and running buffer were raised to twice those in the standard protocol by Laemmli [32] to improve resolution of proteins in low-mass ranges, and proteins were transferred to Invitrolon PVDF membranes using a buffer containing 25 mM glycine, 192 mM Tris-HCl, 10% (v/v) methanol, and 0.01% (v/v) SDS. Membranes were incubated with anti-PrP antibodies, 44B1 [33] or SAF-84 (Cayman Chemical, Ann Arbor, MI, USA) [34, 35], in Can Get Signal-1 immunoreaction enhancer solution (Toyobo Co., Ltd.). After washing with PBS containing 0.05% (v/v) Tween 20, membranes were incubated with horseradish peroxidase-conjugated AffiniPure F(ab’)2 anti-mouse IgG (Jackson ImmunoResearch Laboratories, Inc., PA, USA) or VeriBlot anti-mouse IgG (Abcam plc., Cambridge, UK) in Can Get Signal-2 solution (Toyobo Co., Ltd.). Chemiluminescent signals using Pierce ECL Plus Western blotting substrate (Thermo Fisher Scientific Inc., Thermo Scientific) were detected with Super RX films (Fujifilm, Tokyo, Japan) or a LAS-3000 mini imaging system (Fujifilm) equipped with a charge-coupled device camera (CCD camera). Signal intensities were quantified by ImageGauge software (Fujifilm), and data were fitted to regression curves of one-phase exponential decay using Prism 6 software (GraphPad Software, Inc., La Jolla, CA, USA). Other antibodies used were rabbit anti-glial fibrillary acidic protein (GFAP) (polyclonal, Dako Agilent, Santa Clara, CA, USA, product number: Z0334), rabbit anti-heat shock protein 25 (HSP25) (polyclonal, Stressgen Enzo, Farmingdale, NY, USA, product number: SPA-801), mouse anti-glyceraldehyde-3-phosphate dehydrogenase (GAPDH) (monoclonal, OriGene-Acris Antibodies, Rockville, MD, USA, product number: ACR001PT), and mouse anti-neuronal class III β-tubulin (monoclonal, Covance-BioLegend, San Diego, CA, USA, clone TUJ1). Alkaline phosphatase-conjugated AffiniPure F(ab’)2 anti-rabbit IgG (Jackson ImmunoResearch Laboratories, Inc.) and CDP-star substrate (Applied Biosystems, Thermo Scientific) were used for detection of primary antibodies raised in rabbits.

### Histopathology and immunohistochemistry

Mouse brains, spleens, and distal regions of the ileum were fixed with 20% formalin in PBS, treated with formic acid, and processed as described previously [28, 36]. Tissue sections of 3 μm thickness were mounted on Frontier FRC-11 microscope glass slides (Matsunami Glass Ind., Ltd., Osaka, Japan), and subjected to routine histological staining with hematoxylin and eosin. For immunohistochemistry of PrPSc, antigen retrieval was performed by hydrolytic autoclaving at 121°C for 20 min in 1 mM HCl for brain sections or 3 mM HCl for spleen and the ileum sections. Endogenous peroxidase activity was inactivated by treating sections with 0.3% (v/v) hydrogen peroxide in methanol for 30 min at room temperature. Sections were then incubated with anti-PrP antibody T4 [37] in PBS. After washing sections with PBS, immunopositive signals for PrPSc were detected using the EnVision+ system (Dako Agilent, Santa Clara, CA, USA) with 3, 3'-diaminobenzidine (Dojindo laboratories, Kumamoto, Japan) as a substrate. For GFAP immunohistochemistry, sections were processed on the glass slides in citrate buffer (pH 6.0) at 121°C for 10 min for antigen retrieval according to a standard immunohistochemical procedure. GFAP was detected using an anti-GFAP antibody (Dako Agilent, product number: Z0334). Specimens were observed using UPlan Apo objective lenses (Olympus, Tokyo, Japan) and photographed with a DP21 digital camera (Olympus).

## Results

### Transmission of bovine C- and L-BSE prions to primates reduced differences in biochemical traits of PrPSc

**Fig 1:**
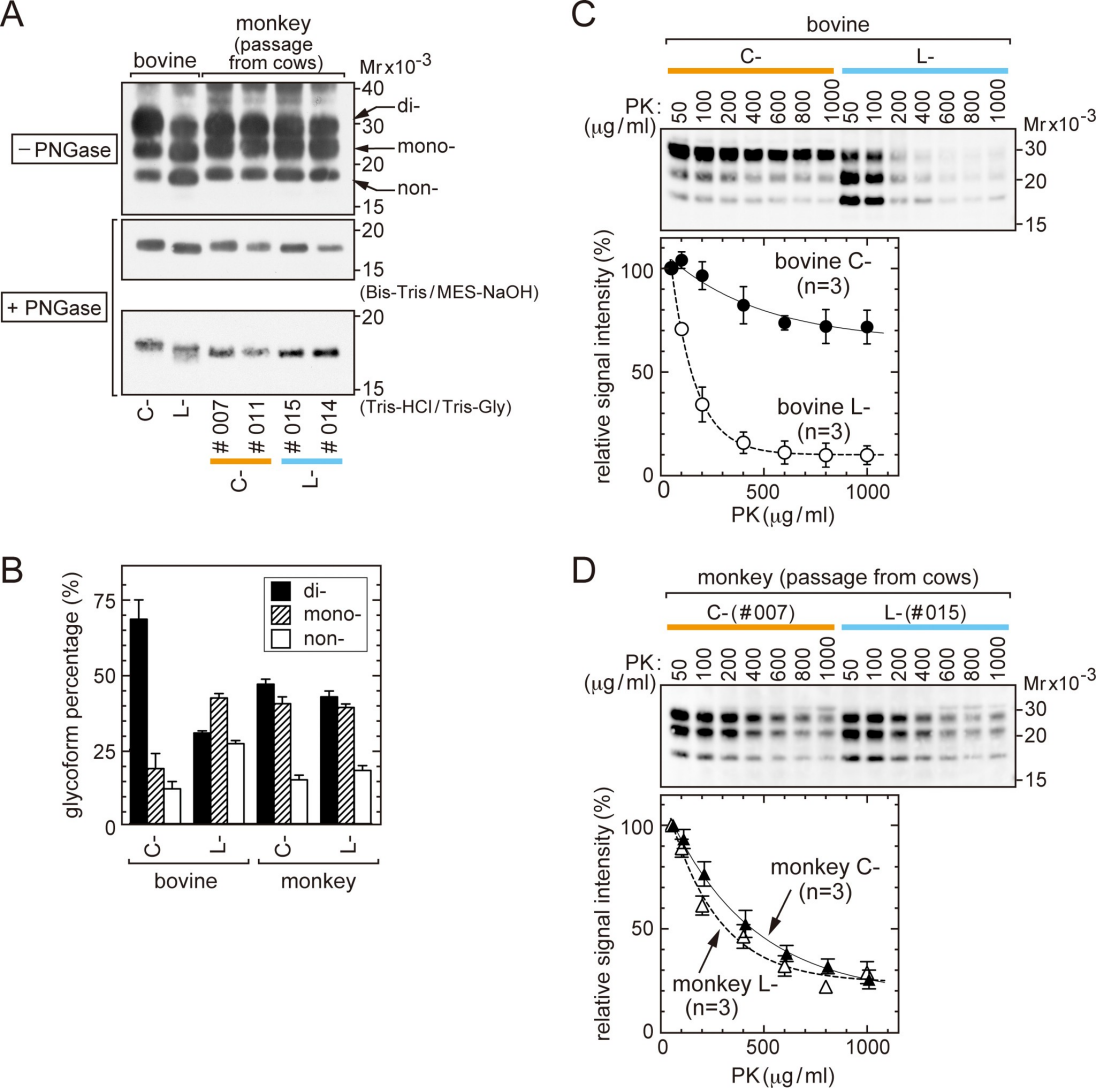
Biochemical traits of PrPSc of C- and L-BSE prions accumulated in the brains. (A) Western blot analysis. Homogenates of bovine and monkey brains were digested by PK, and subjected to SDS-PAGE using a Bis-Tris/MES-NaOH buffer system (top panel). Alternatively, PK digests were further treated by PNGase F, and subjected to SDS-PAGE using a Bis-Tris/MES-NaOH buffer system (middle) or a Tris-HCl/Tris-glycine buffer system (bottom). Monkeys #007 and #011 were infected by bovine C-BSE prions; monkeys #014 and #015 were infected by bovine L-BSE prions. Mr; Relative molecular mass. (B) Ratios of signal intensities of di-, mono-, and non-glycosylated forms of PrPSc by Western blot analysis. PrPSc was detected using a LAS-3000 mini imaging system. Data shown are from four independent analyses. (C) Western blot analysis showing PK sensitivity of PrPSc of bovine C- and L-BSE prions. Brain homogenates were incubated with PK at the indicated concentrations (37°C for 30 min). PrPSc was detected using the LAS-3000 mini imaging system, and signal intensities of PrPSc were plotted relative to those detected in samples digested with 50 μg/mL of PK, which were set at 100.0%. The data are from three independent analyses. (D) PK sensitivity of PrPSc of monkey C- and L-BSE prions, as in (C). The data are from three independent analyses of homogenates of the brains of #007 and #015. In (A), (C), and (D), the anti-PrP antibody SAF-84 was used.

C- and L-BSE prions isolated from cattle brains (referred to here as bovine C- and L-BSE prions) exhibited the following differences: i) different migration of each non-glycosylated PrPSc on SDS-PAGE [10, 38, 39], ii) different abundance ratios of di-, mono-, and non-glycosylated PrPSc [10, 29, 38, 39], iii) different degrees of resistance to digestion by PK [38, 40], and iv) different pathological phenotypes in the brains of affected cows [10, 29, 39]. However, in experimental transmission to cynomolgus monkeys of bovine C-BSE prions (monkey identification numbers (ID numbers) #007, #010, and #011) [23] and bovine L-BSE prions (ID numbers #014 and #015) [17], the two prions after propagation (referred to here as monkey C- and L-BSE prions) displayed only subtle SDS-PAGE migration differences in PrPSc from PK-digested monkey brain homogenates using a Bis-Tris/MES buffer system (Fig 1A, top and middle panels) or a Tris-HCl/Tris-glycine buffer system (Fig 1A, bottom panel). In addition to similar electrophoretic migration, monkey C- and L-BSE prions exhibited comparable PrPSc glycoform profiles (Fig 1A, top panel; Fig 1B).

We further examined the resistance of each prion to digestion by PK. As reported previously, PrPSc of bovine C-BSE prions exhibited a higher resistance to PK than bovine L-BSE prions in a range of PK concentrations from 50 μg/mL to 1000 μg/mL, yielding sustained signals of PrPSc by Western blot analysis (Fig 1C) [38, 40]. By contrast, PrPSc of monkey C- and L-BSE prions had moderately reduced resistance to PK, and the magnitudes of resistance were indistinguishable (Fig 1D).

In aggregate, PrPSc of monkey C- and L-BSE prions had similar biochemical traits, as demonstrated by electrophoretic migration profiles, glycoform profiles, and resistance to PK digestion.

### Bovine L-BSE prions were not transmissible to inbred mice in the first or second challenge

A previous study of transmission of bovine C-BSE prions, vCJD prions, and monkey C-BSE prions to C57BL/6 mice demonstrated corresponding pathological profiles in the mouse brains, which provided corroborative evidence of the etiological link between bovine C-BSE and vCJD [15]. With this in mind, we first examined the transmissibility of bovine L-BSE prions to three lines of inbred mice, C57BL/6, SJL, and RIIIS.

**Fig 2:**
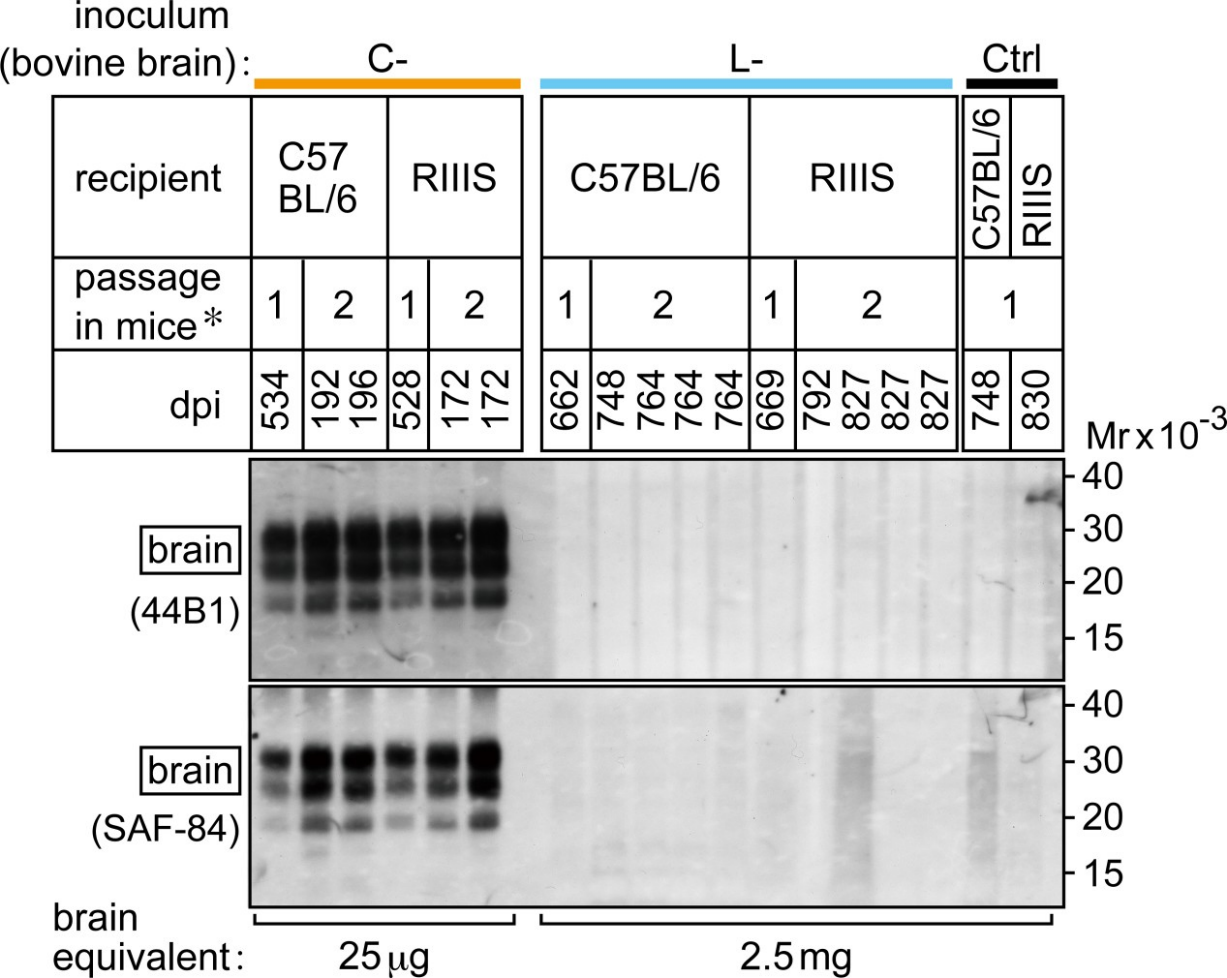
PrPSc in the brains of C57BL/6 and RIIIS mice challenged by bovine C- and L-BSE prions. Ctrl; saline. dpi; days post-inoculation. *Numbers indicate the first (1) and second (2) passages. In the second passage, mouse brain homogenates obtained in the first passage were injected intracranially into the same mouse line. Brains were digested with PK and subjected to analysis. The antibodies, 44B1 or SAF-84, and VeriBlot anti-mouse IgG were used to detect PrPSc. Representative mice challenged by C-BSE prions (each sample equivalent to 25 μg of brain), or L-BSE prions, and Ctrl mice (equivalent to 2.5 mg of brain) are shown. Mr; relative molecular mass.

Mice were inoculated intracranially with a brain homogenate from a C-BSE-affected cow or from an L-BSE-affected cow. The inocula contained equivalent amounts of PrPSc (S1 Fig). First passage inoculation with bovine C-BSE prions caused disease in mice (Table 1). Mice inoculated with bovine L-BSE prions showed no signs of disease throughout their lifetime in the first challenge. PrPSc was not detected in the brains of these mice by Western blot analysis using the anti-PrP antibodies 44B1 [33] and SAF-84 [34, 35] (Fig 2). The spleens and distal regions of the ileum of these mice were also negative for PrPSc by Western blot analysis (Table 1, S2 Fig). By histopathological examination and immunohistochemical staining of PrPSc, the brains of mice inoculated with bovine L-BSE prions were also negative for spongiosis and accumulation of PrPSc (Table 1, S2 Fig).

**Table 1:** Transmission of bovine C- and L-BSE prions to C57BL/6, SJL, and RIIIS mice.

Inoculum	Passage	Recipient mouse	Survival days (dpi)^a^ of affected mice	Affected^b^ / Total no. mice	Mean dpi ± SEM
C-BSE prions	1st; cow to mouse	C57BL/6	511, 534, 567, 652, 662, 704, 729, 729, 729	9 / 11	646 ± 29
		SJL	262, 269, 304, 316, 368, 373, 393, 408, 408	9 / 10	345 ± 19^c^
		RIIIS	430, 528, 574, 606, 669	5 / 5	561 ± 40
	2nd; mouse to mouse^c^	C57BL/6	175, 184, 186, 192, 192, 192, 196	7 / 7	188 ± 3
		SJL	141, 161, 172, 179, 181, 181, 181, 184, 184, 189	10 / 10	174 ±5
		RIIIS	168, 172, 172, 172	4 / 4	171 ± 1
	3rd; mouse to mouse^c^	C57BL/6	174, 187, 187, 187, 187, 188, 189	7 / 7	186 ± 2
L-BSE prions	1st; cow to mouse	C57BL/6	– (> 813)^d^	0 / 10	–
		SJL^e^ [41]	– (> 408)	0 / 10	–
		RIIIS	– (> 850)	0 / 6	–
	2nd; mouse to mouse^c^	C57BL/6	– (> 764)	0 / 12	–
		SJL^e^ [41]	– (> 591)	0 / 10	–
		RIIIS	– (> 827)	0 / 4	–

^a^Humane endpoints (dpi; days post-inoculation).

^b^Number of mice exhibiting positive signals for PrP^Sc^ by Western blot analysis of brain, spleen, or distal ileum.

^c^Brain homogenates were inoculated into the same mouse lines.

^d^Mice did not develop the disease by the dpi shown in parentheses. Western blot analysis and immunohistochemistry were negative for PrP^Sc^, and histological examination did not demonstrate any disease-associated neuropathological changes.

^e^Sacrificed before reticulum cell sarcoma developed, which is known to occur spontaneously in SJL mice due to aging.

In the second passage (i.e., mouse-to-mouse passage), brain homogenates from single C57BL/6, SJL, or RIIIS mice at the terminal stage of first passage infection with bovine C-BSE prions were inoculated into the same mouse line. Subsequently, all mice developed the disease in a shortened period (Table 1). In a parallel experiment, pooled brain homogenates prepared from asymptomatic mice from the first challenge with bovine L-BSE prions (the brains of three C57BL/6 mice collected 662 days, two SJL mice collected 408 days, and four RIIIS mice collected 669 days post-inoculation) were injected intracranially into the same mouse lines. None of the mice developed signs of neurological disease. PrPSc was not detectable in the brains (Fig 2), spleens, or distal regions of the ileum (Table 1).

### L-BSE prions after propagation in monkeys were not transmissible to C57BL/6 mice in the first or second challenge

**Fig 3:**
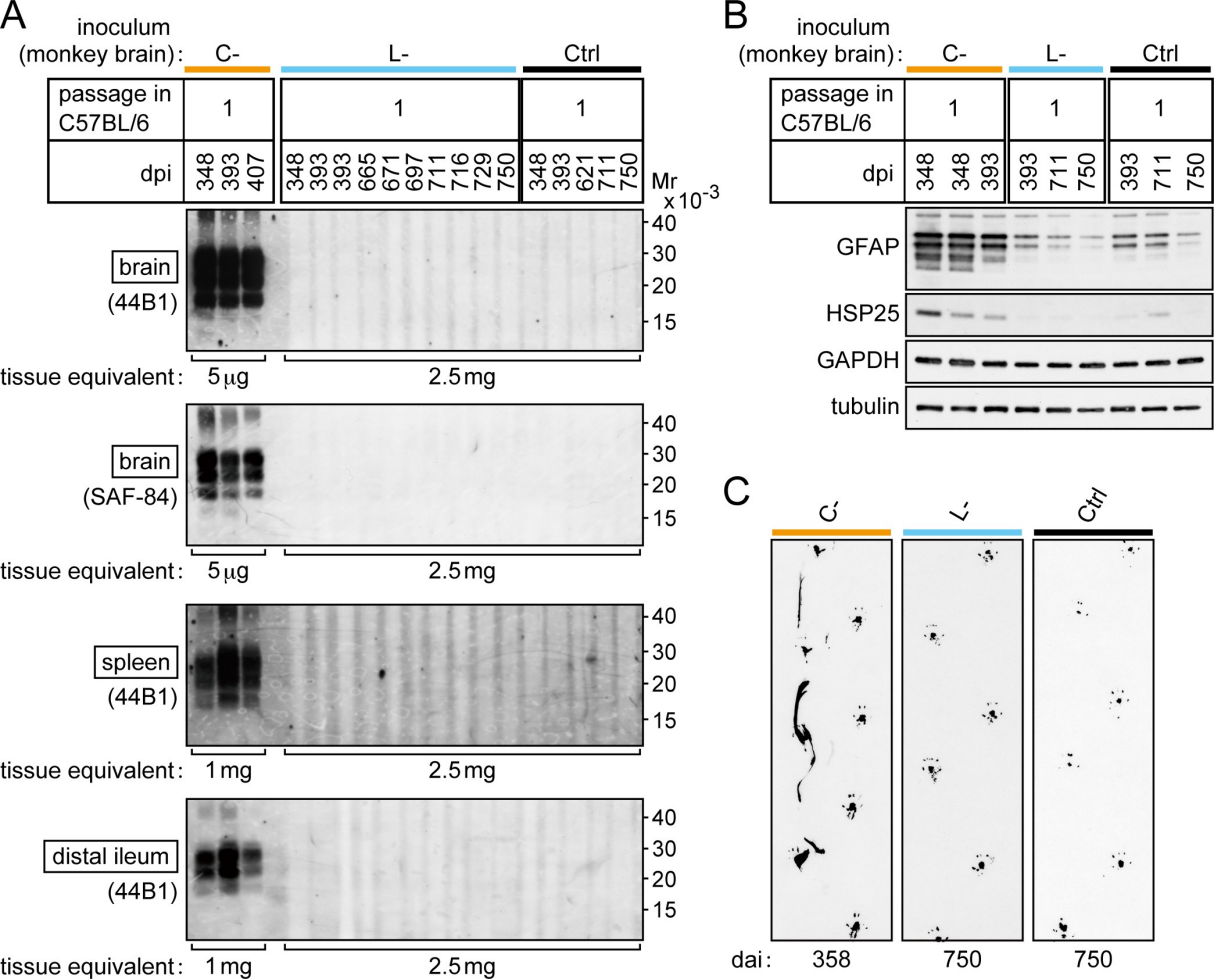
First challenge of C57BL/6 mice with monkey C- and L-BSE prions. (A) Western blot analysis of PrPSc in the brain, spleen, and distal region of the ileum of representative mice. Ctrl (control inoculum); brain homogenates of a healthy monkey. dpi; days post-inoculation. Mr; relative molecular mass. PrPSc was detected using the antibodies 44B1 or SAF-84, and VeriBlot anti-mouse IgG. (B) Western blot analysis of levels of GFAP and HSP25 in the brains of mice. GAPDH and neuronal class III β-tubulin (tubulin) were used as loading controls. (C) Posterior footprints of representative mice subjected to first challenge with monkey C- and L-BSE prions, and a mouse injected with control inoculum (Ctrl as in (A)). The posterior limbs were dipped in India ink, and mice were allowed to walk on sheets of paper.

**Fig 4:**
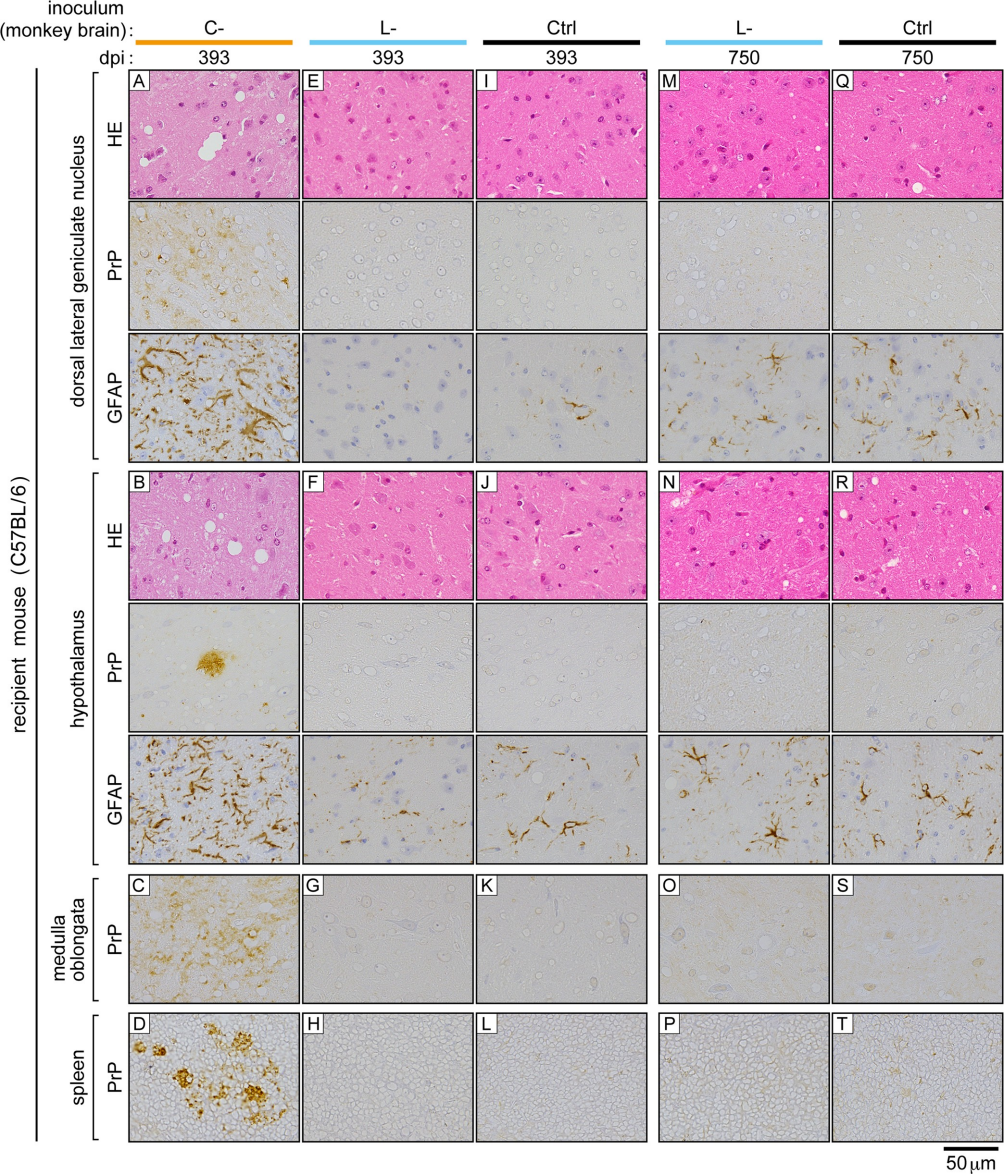
Brains and spleens of C57BL/6 mice subjected to first challenge with monkey C- and L-BSE prions. The brain (corresponding to dorsal lateral geniculate nucleus, hypothalamus, and ventral portion of the medulla oblongata) and spleen (corresponding to the white pulp) are shown. Ctrl (control inoculum); brain homogenates of a healthy monkey. dpi; days post-inoculation. HE; hematoxylin-eosin staining. PrP; immunohistochemical staining of PrPSc. GFAP; immunohistochemical staining of GFAP. The scale bar applies to all panels.

**Fig 5:**
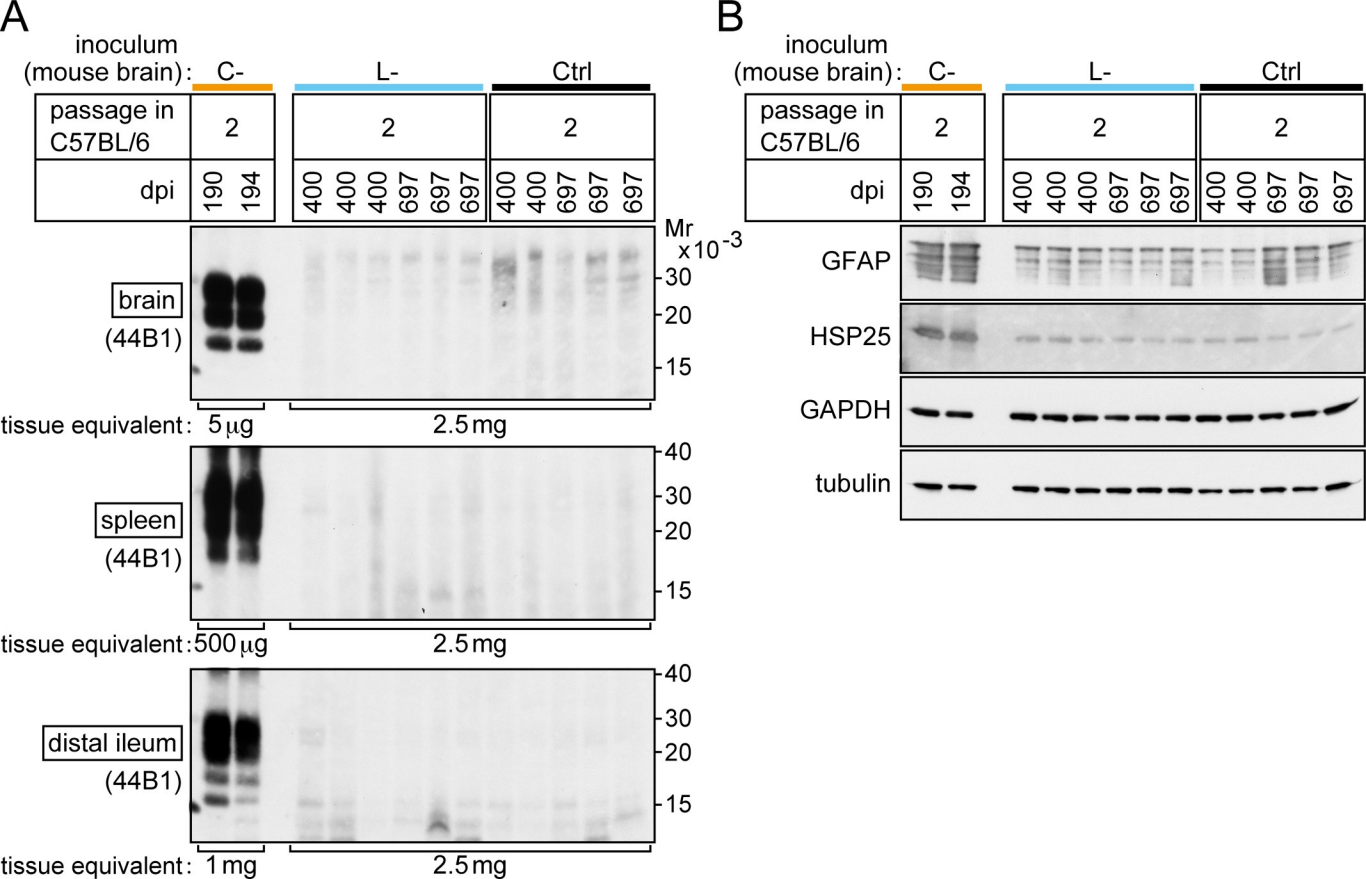
Secondary challenge of C57BL/6 mice with monkey C- and L-BSE prions after passage through C57BL/6 mice. (A) Western blot analysis of the brain, spleen, and distal region of the ileum of representative mice. Ctrl (control inoculum); brain homogenates from a mouse that received brain homogenate from a healthy monkey in the first challenge. dpi; days post-inoculation. Mr; relative molecular mass. PK-digests equivalent to the tissue weights indicated at the bottom of each panel were examined. PrPSc was detected using the antibody 44B1 and VeriBlot anti-mouse IgG. (B) Western blot analysis of GFAP and HSP25 in the brains of mice. GAPDH and tubulin were used as loading controls.

**Fig 6:**
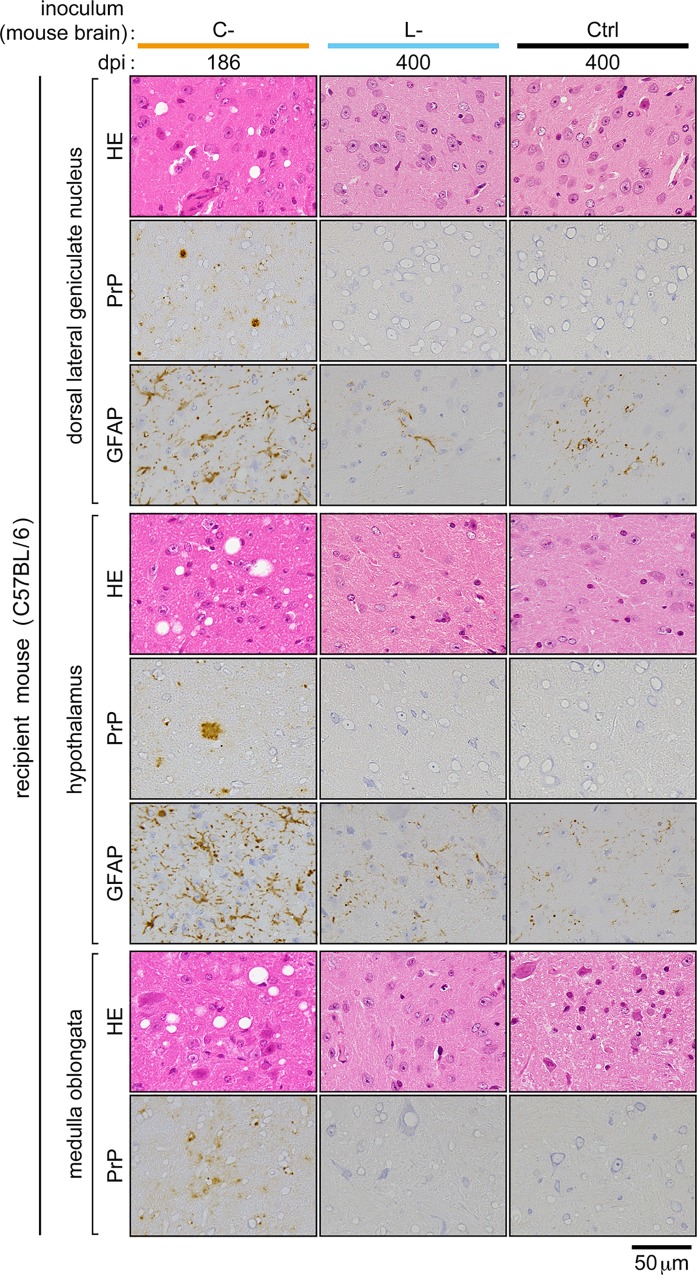
Brains of C57BL/6 mice subjected to secondary mouse-to-mouse challenge with monkey C- and L-BSE prions. Regions of each brain corresponding to the dorsal lateral geniculate nucleus, hypothalamus, and ventral portion of the medulla oblongata are shown. Ctrl (control inoculum); brain homogenates from a mouse that received the brain homogenate of a healthy monkey in the first challenge. dpi; days post-inoculation. HE; hematoxylin-eosin staining. PrP; immunohistochemical staining of PrPSc. GFAP; immunohistochemical staining of GFAP. The scale bar applies to all panels.

The clear difference in the transmissibility of bovine C- and L-BSE prions to inbred mice enabled us to clarify the identity of monkey L-BSE prions using a mouse bioassay. The brain homogenate of a representative monkey (ID number #007) with the disease by bovine C-BSE prions, and the brain homogenate of a representative monkey (ID number #015) with the disease by bovine L-BSE prions were inoculated to C57BL/6 mice. The inocula contained an equivalent amount of PrPSc (S1 Fig). Consequently, mice that received monkey C-BSE prions developed the disease in the first passage (Table 2), as previously reported [15]. PrPSc was detectable in the brain, spleen, and distal regions of the ileum by Western blot analysis (Fig 3A). Furthermore, increased amounts of GFAP and HSP25 were detected in the brain (Fig 3B), as reported in a murine model of BSE [42]. Histopathological examination and immunohistochemical staining of PrPSc in the brain revealed spongiosis and dense accumulation of PrPSc in the thalamus, particularly in the lateral posterior nucleus of the thalamus, lateral geniculate nuclei, hypothalamic lateral zone, and medulla oblongata (Fig 4, panels A, B, and C). In these lesions, GFAP expression was increased compared to that in mice that received monkey L-BSE prions (Fig 4, panels E, F, G, M, N, and O) and control mice (Fig 4, panels I, J, K, Q, R, and S). In spleen immunohistochemistry, PrPSc was detected in regions of white pulp (Fig 4, panel D). In the second passage of monkey C-BSE prions in C57BL/6 mice, the incubation period was shortened to 189 ± 1 days (Table 2). PrPSc accumulated in the brain, spleen, and distal regions of the ileum (Fig 5). Severe spongiosis was observed in the brain, and immunohistochemical staining of PrPSc revealed accumulation of PrPSc in the brain, particularly in the lateral posterior nucleus of the thalamus, lateral geniculate nuclei, hypothalamic lateral zone, and medulla oblongata (Fig 6), as observed in the first passage mice inoculated with monkey brain homogenate (Fig 4).

**Table 2:** Transmission of monkey-passaged C- and L-BSE prions to C57BL/6 mice.

Inoculum	Passage	Survival days (dpi)^a^ of affected mice	Affected^b^ / Total no. mice	Mean dpi ± SEM
Monkey C-BSE prions	1st; monkey to C57BL/6	345, 348, 348, 370, 372, 375, 379, 393, 401, 407, 407	11 / 11	377 ± 7
	2nd; C57BL/6 to C57BL/6	185, 186, 186, 188, 190, 190, 193, 194	8 / 8	189 ± 1
Monkey L-BSE prions	1st; monkey to C57BL/6	– (> 750)^c^	0 / 12	–
	2nd; C57BL/6 to C57BL/6	– (> 697)	0 / 10	–
Control (Brain homogenate of a healthy monkey)	1st; monkey to C57BL/6	– (> 750)	0 / 8	–
	2nd; C57BL/6 to C57BL/6	– (> 697)	0 / 8	–

^a^Humane endpoints (dpi; days post-inoculation).

^b^Number of mice exhibiting positive signals for PrP^Sc^ by Western blot analysis of brain, spleen, or distal ileum.

^c^Mice did not develop the disease by the dpi shown in parentheses. Western blot analysis and immunohistochemistry were negative for PrP^Sc^, and histological examination did not demonstrate any disease-associated neuropathological changes.

By contrast, inoculation with monkey L-BSE prions did not induce neurological signs of disease such as ataxia in mice (Table 2, Fig 3C). PrPSc was not detectable in the brain, spleen, or distal regions of the ileum by Western blot analysis even at 750 days post-challenge (Fig 3A). Histopathological and immunohistochemical examination of the brains did not reveal any pathological changes or deposition of PrPSc, similar to the brains of mice challenged with the brain homogenate of a healthy monkey (Ctrl) (Fig 4). In the brains of long-lived mice challenged with monkey L-BSE prions, we observed sparse vacuoles (Fig 4, panels M and N). Such vacuoles were, however, found at a similar frequency in age-matched control mice (Fig 4, panels Q and R). Thus, these vacuoles were considered to be due to aging and not the disease. The amounts of GFAP and HSP25 in the brain were at basal levels in these mice (Fig 3B, Fig 4).

**Fig 7:**
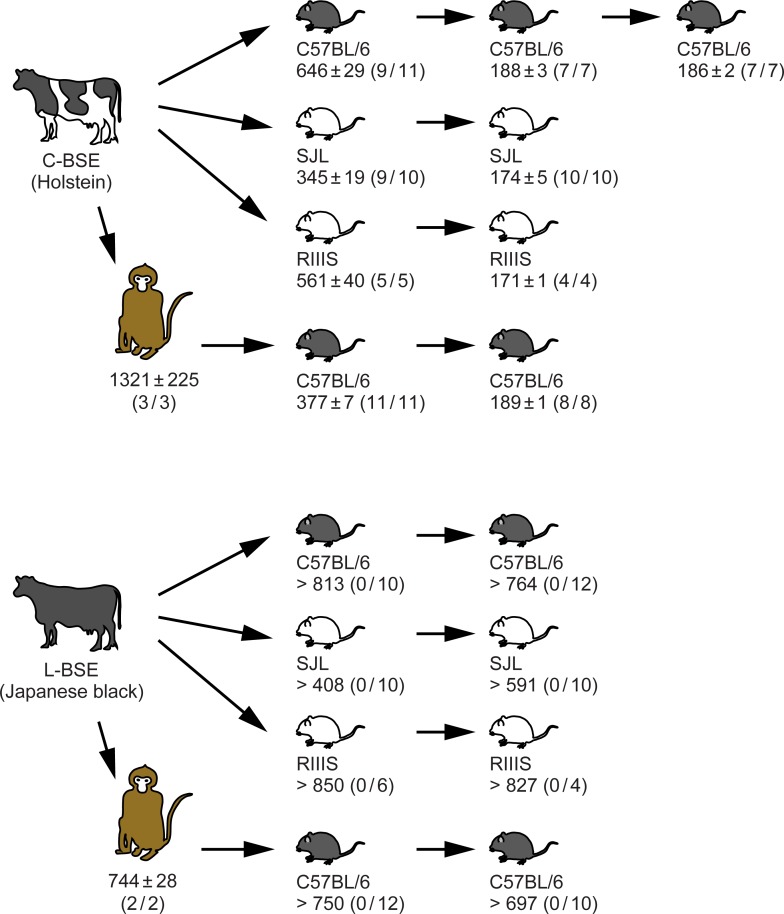
Flow chart of the transmission scheme and results. Mean endpoints in days post-inoculation ± SEM are shown. Numbers in parentheses are: ‘the number of mice positive for PrPSc in the brain, spleen, or ileum by Western blot analysis’ divided by ‘number of mice in the group’. Data for transmission of bovine C- and L-BSE prions to cynomolgus monkeys are from our previous studies [17, 23].

Homogenates of the pooled brains of asymptomatic mice (three mice sacrificed 711, 716, and 750 days post-inoculation with monkey L-BSE prions) were used for second passage intracranial injections into C57BL/6 mice to amplify PrPSc [24, 43], as it was undetectable in the first challenge. By contrast to the secondary mouse-to-mouse passage of monkey C-BSE prions, the secondary mouse-to-mouse passage of monkey L-BSE prions induced no signs of disease by 680 days post-injection, and PrPSc was undetectable in the brain, spleen, and distal regions of the ileum by Western blot analysis (Fig 5A). Amounts of GFAP and HSP25 in the brain were normal (Fig 5B). Neither spongiosis nor increased expression of GFAP was observed in histopathological and immunohistochemical examinations of the brains (Fig 6). Fig 7 is a flow chart that summarizes the results shown in Tables 1 and 2.

### Transmission of bovine C-BSE prions to monkeys did not alter their pathogenicity in C57BL/6 mice

**Fig 8:**
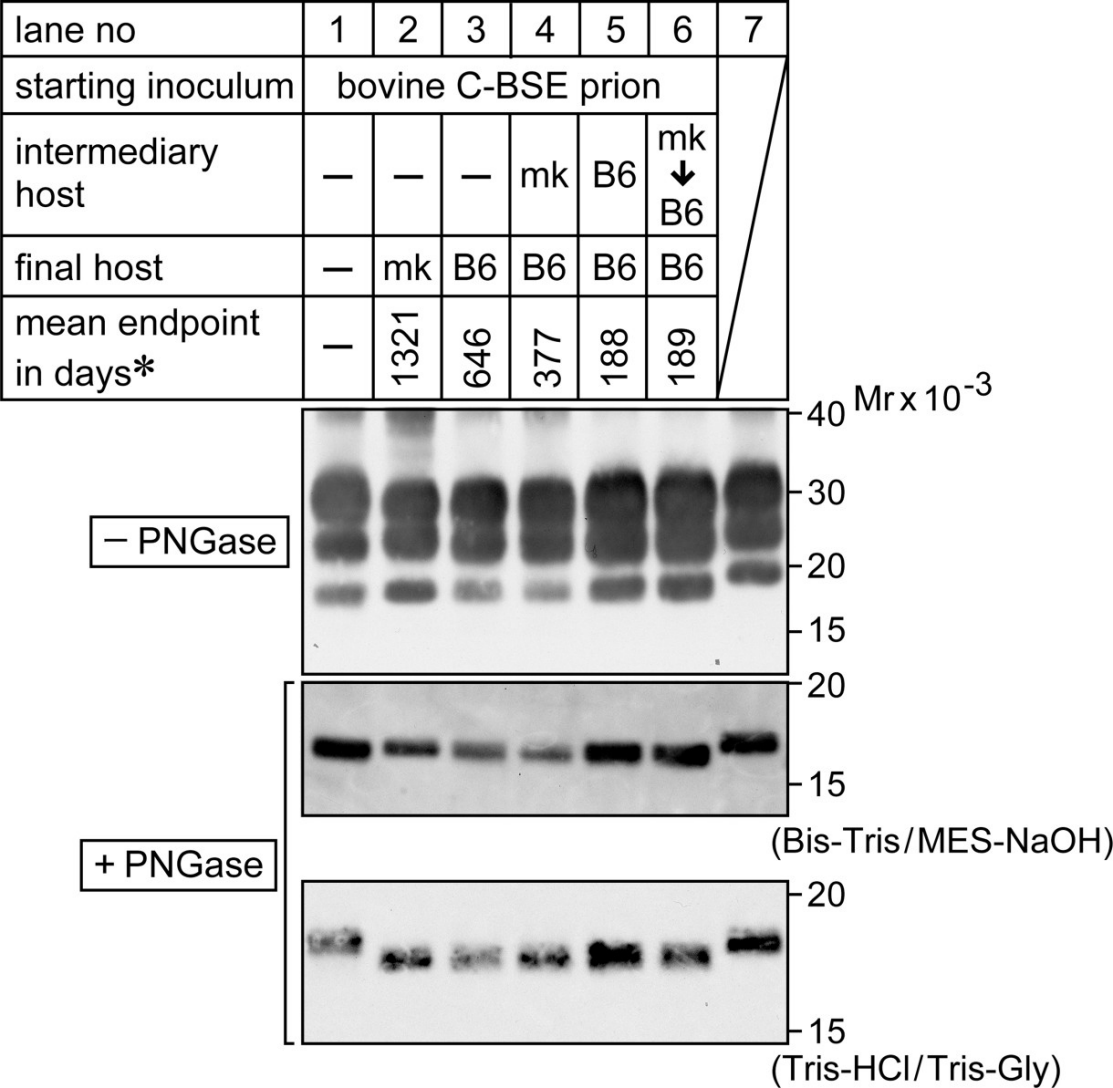
Electrophoretic profiles of PrPSc of C-BSE prions that accumulated in the brain. Lane 1; bovine C-BSE prions, lane 2; C-BSE prions after passage in cynomolgus monkeys (indicated as mk), lane 3; C-BSE prions after first passage in C57BL/6 mice (indicated as B6), lane 4; after passage in mk, then in B6, lane 5; after two serial passages in B6, lane 6; after passage in mk, then two serial passages in B6. Lane 7 shows PrPSc of scrapie prion (Obihiro I strain) after adaptation to B6. Aliquots of PK-digests of brains were subjected to SDS-PAGE before (top panel) or after treatment with PNGase F (middle and bottom panels). Electrophoresis was carried out in Bis-Tris/MES-NaOH buffer (top and middle) or in Tris-HCl/Tris-glycine buffer (bottom). PrPSc was detected using the antibody SAF-84. *; the mean endpoint in days shown in Fig 7. Mr; relative molecular mass.

The first passage of bovine C-BSE prions to C57BL/6 mice resulted in an endpoint of 646 ± 29 days (the mean ± SEM, n = 11) (Table 1, Fig 6), whereas the first passage of monkey C-BSE prions to C57BL/6 mice resulted in an endpoint of 377 ± 7 days (n = 11) (Table 2, Fig 6). A similar gap in endpoints of bovine C- and the monkey C-BSE prions in C57BL/6 mice in the first passage was reported in a previous study, in which the authors reasoned it to be due to different titers of prions in the inocula [15]. Indeed, the inoculum of monkey C-BSE prions in our experiment had a higher concentration of PrPSc than that of bovine C-BSE prions (S1 Fig, panel E). However, as bovine C-BSE prions and monkey C-BSE prions had different biochemical traits based on electrophoretic migration, and different glycoform profiles of PrPSc (Fig 1A; Fig 8, lanes 1 and 2), it is possible that transmission of C-BSE prions to monkeys altered the C-BSE prions and increased their virulence in mice. To clarify this point, we carried out a secondary mouse-to-mouse transmission experiment. The endpoint observed for mouse-to-mouse transmission of bovine C-BSE prions was 188 ± 3 days (mean ± SEM, n = 7) (Table 1, Fig 6), consistent with that for mouse-to-mouse transmission of monkey C-BSE prions (189 ± 1 days, mean ± SEM, n = 8) (Table 2, Fig 6). This result negated the possibility of alteration of C-BSE prions by transmission to monkeys. We also suspect that different magnitudes of species barrier to mice from bovine C-BSE prions or monkey C-BSE prions contribute to the gap.

As mentioned above, bovine C- and monkey C-BSE prions had differences in their PrPSc electrophoretic migration and glycoform profiles. However, after passage of bovine C- and monkey C-BSE prions to C57BL/6 mice, the electrophoretic profiles of the PrPSc that accumulated in the mouse brain were indistinguishable (Fig 8, compare lanes 3 and 4 for the first passage to mice, and lanes 5 and 6 for the second passage to mice). These PrPSc profiles were specific to bovine and monkey C-BSE prions. They did not match the PrPSc profile of scrapie prions (Obihiro I strain) adapted to C57BL/6 mice (Fig 8, lane 7) [44].

## Discussion

Interspecies prion transmission entails adaptation of the original prions to a new host animal. During this process, prion strain traits can be retained or unpredictably modified. Studies to date have reported that experimental transmission of C-, L-, and H-BSE prions from cows to other animals has prompted emergence of different strain types. Examples have been found in transmission of C-BSE prions to inbred mice (SJL) [45], to transgenic mice expressing human PrPC [46], to transgenic mice expressing mouse-hamster chimeric PrPC [47], and to sheep [48]. Likewise, transmission of H-BSE prions to inbred mice or transgenic mice expressing bovine PrPC caused emergence of the C-BSE prion phenotype(s) [49–51]. L-BSE prion transmission to inbred mice (discussed below) [24] or to transgenic mice expressing sheep PrPC [19] generated prions that were biochemically and pathogenetically similar to C-BSE prions. In addition, transmission of L-BSE prions to sheep induced the emergence of prions that possessed altered pathogenicity [25] or induced an unknown phenotype [26].

The zoonotic potential of L-BSE prions is suggested by transmissibility from cows to cynomolgus monkeys (*Macaca fascicularis*) [16, 17] and lemurs (*Microcebus murinus*) [18]. The brains of cynomolgus monkeys infected with C- or L-BSE prions exhibited different pathological phenotypes [13, 16, 17, 23], whereas each type of PrPSc that accumulated in the brain had similar biochemical traits (Fig 1). Considering the plasticity of L-BSE prions during interspecies transmission in previous studies, the potential for L-BSE prions in a non-human primate model to lead to emergence of a C-BSE strain type was of concern because such a phenomenon challenges our current understanding of the etiology of vCJD. However, we found no evidence in the present study to support this. Recently, an *in vitro* real-time quaking-induced conversion (RT-QuIC) assay was applied to brain samples from cynomolgus monkeys infected with C- and L-BSE prions from cows [52]. In the reported data of this RT-QuIC assay, signals unique to C-BSE prions were not observed in brain samples from monkeys infected with L-BSE prions. These data suggest that infection of cynomolgus monkeys with L-BSE prions did not induce C-BSE prions in the brain above the detection limit of the RT-QuIC assay. Our bioassay results support this. From these results, we suspect the possibility of L-BSE prions transforming into C-BSE prions in humans is low, although the parallelism of monkeys and humans should be carefully considered.

In terms of genetic polymorphism, the codon corresponding to PrPC amino acid position 129 encodes methionine or valine in human *Prnp*. The cynomolgus monkeys in the present study had *Prnp* with the 129Met/Met genotype, corresponding with the human 129Met/Met genotype (see Materials and methods). The polymorphism at codon 129 is known to affect the phenotypes of sporadic CJD patients [1] and to determine human susceptibility to C-BSE prions causing vCJD (reviewed in [53]). As no case of human infection by L-BSE prions has been reported, the effects of the polymorphism at codon 129 on human susceptibility to L-BSE prions are currently unclear. To address this question, brain tissues from transgenic mice with human *Prnp* homozygous for methionine (129Met/Met) or homozygous for valine (129Val/Val) were assessed by *in vitro* protein misfolding cyclic amplification (PMCA) analysis. PMCA analysis revealed that PrPC with 129Met/Met and 129Val/Val genotypes could both be converted to PrPSc *in vitro* using bovine L-BSE prions as seeds [54].

In a prior study, L-BSE prions that accumulated in the bovine brain were compared with a panel of sporadic CJD prions by transmission to transgenic mice expressing human PrPC (tg650 mice). L-BSE prions elicited a 100% attack rate in tg650 mice, but no evidence was found to support a link between bovine L-BSE prions and known types of sporadic CJD prions [55]. In another study, L-BSE prions that had been transmitted from cows to cynomolgus monkeys were inoculated into transgenic mice expressing bovine PrPC (tg100 mice). They reported that monkey L-BSE prions induced disease in tg100 mice with a 100% attack rate, suggesting that propagation of L-BSE prions in cynomolgus monkeys does not attenuate its virulence in cattle [56]. The present study demonstrates the preserved virulence of bovine and monkey L-BSE prions, by making use of inbred mice that were permissive for C- but not for L-BSE prions. The results of the present study and those of the studies on the tg650 and the tg100 mice are complementary, and are important for understanding the nature of the L-BSE prion agent.

Lastly, we confirmed that bovine L-BSE prions were not transmissible to three lines of inbred mice by intracranial administration (Table 1). In another study, L-BSE prions were reported to develop into a prion agent with similar signatures as bovine C-BSE prions after two serial passages from cows to C57BL/6 or SJL mice [24]. The reasons for these inconsistent results are unknown, but one possibility is different routes of inoculation into mice, as noted previously [25]; intracranial inoculation was employed in our study, whereas a combination of intracerebral and intraperitoneal inoculations was used for the first passage in the previous report [24]. On this subject, routes of inoculation have been reported to influence phenotypic features in experimental transmission of C-BSE prions from cows to IM mice (*Prnpb* mice), although strain properties were retained overall [57]. Based on the assumption that prions contain a mixture of components, including a major component, and a quasispecies consisting of a variety of minor components with subtly different conformations [9], it is of interest to investigate whether different routes of inoculation favor propagation of different components within the quasispecies, and if such a selection bias accounts for the distinct phenotypic features in these experiments.

The present study suggests that, although C- and L-BSE prions that propagated in cynomolgus monkeys exhibit similar biochemical PrPSc signatures, composed of the monkey amino acid sequence, the two prions do not merge but retain unique pathogenic traits, possibly by holding strain-specific conformations of PrPSc. We are currently carrying out a mouse bioassay of ‘monkey-to-monkey’-passaged L-BSE prions (i.e., passaged twice in cynomolgus monkeys). L-BSE prions after two passages in the monkeys induced no neurological signs in the mice by 580 days after the inoculation. This study is ongoing and will be reported separately.

## Supporting information

S1 Fig Relative amounts of PrP^Sc^ in inocula injected into C57BL/6 mice. (PDF)

S2 Fig First challenge of C57BL/6 mice with bovine C- and L-BSE prions. (PDF)
