# Supplementary material for: Tracking and clarifying differential traits of classical- and atypical L-type bovine spongiform encephalopathy prions after transmission from cattle to cynomolgus monkeys
Source: PLoS One. 2019 May 16;14(5):e0216807. doi: 10.1371/journal.pone.0216807 (PMC6522098; doi:10.1371/journal.pone.0216807)
Supplement: S1 Fig — (PDF) [file pone.0216807.s001.pdf]

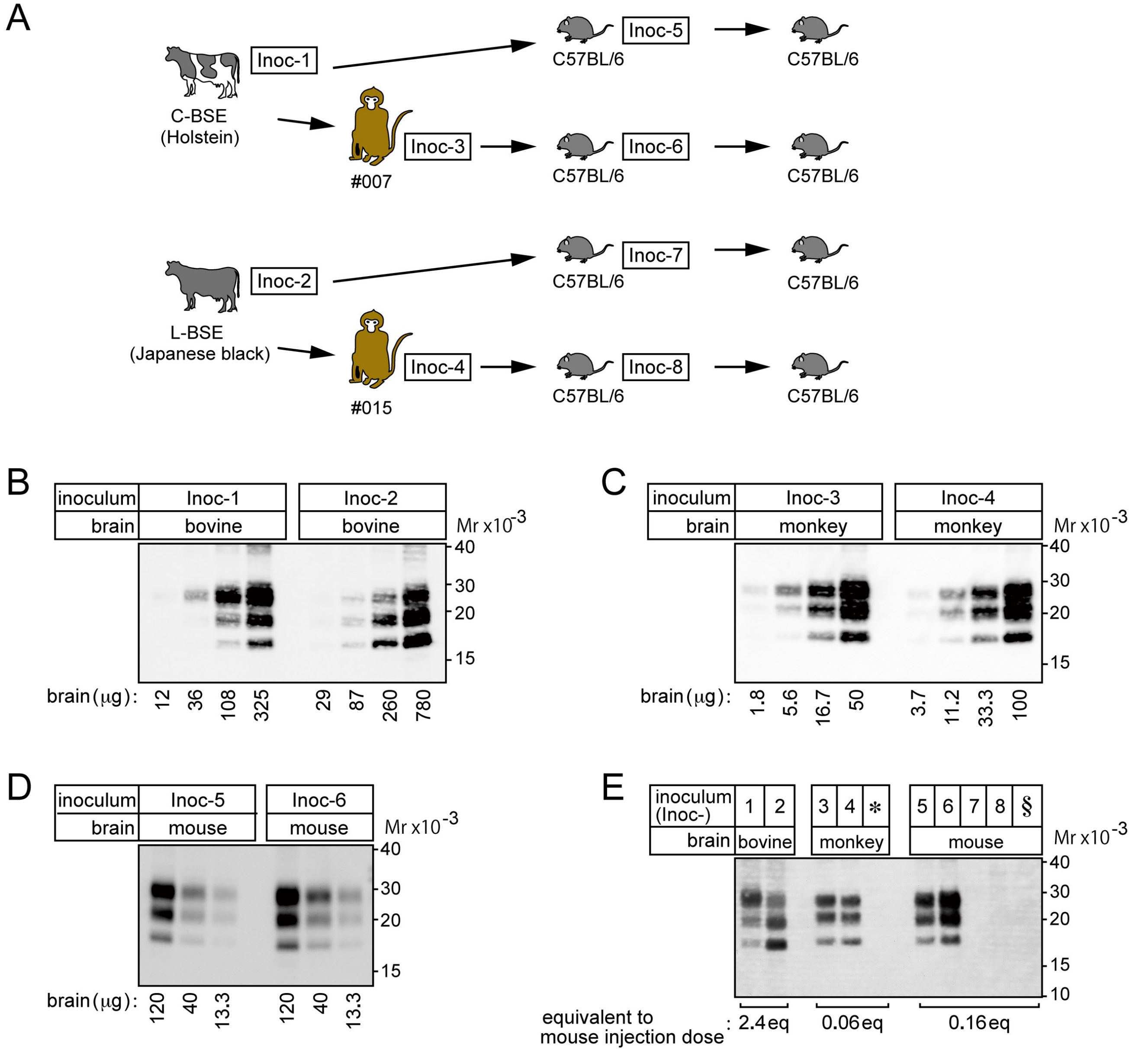

S1 Fig. Relative amounts of PrP^{Sc} in inocula injected into C57BL/6 mice.

(A) Flow chart of bioassays using C57BL/6 mice. Eight types of inocula (Inoc-1 to -8) were prepared and injected into C57BL/6 mice. (B) Western blot analysis of homogenates of bovine brains from which Inoc-1 and -2 were prepared. Brain homogenates were digested by PK, and digests corresponding to indicated weights of brain tissue were analyzed by Western blot to estimate amounts of PrP^{Sc}. (C) Homogenates of monkey brains used as sources of Inoc-3 and -4. Western blot analysis was carried out as in (B). (D) Homogenates of mouse brains used as sources of Inoc-5 and -6. Western blot analysis was carried out as in (B). (E) Comparison of relative amounts of PrP^{Sc} in inocula. After digestion of inocula by PK, digest amounts equivalent to indicated mouse injection doses (Inoc-1 and -2, 2.4x-dose; Inoc-3 and -4, 0.06x-dose; Inoc-5, -6, -7 and-8, 0.16x-dose) were analyzed by Western blot. Comparable PrP^{Sc} signal intensities were obtained for Inoc-1 through -6. PrP^{Sc} was not detectable in Inoc-7 and -8. *; brain homogenate of a healthy monkey (control). §; brain homogenate of a healthy mouse (control). Mr; relative molecular mass. The antibody SAF84 and VeriBlot anti-mouse IgG were used to detect PrP^{Sc} in panels (B) to (E). Inoc-1, -2, -3, and -4 were also injected into SJL and RIIIS mice in this study.
