# Supplementary material for: Tracking and clarifying differential traits of classical- and atypical L-type bovine spongiform encephalopathy prions after transmission from cattle to cynomolgus monkeys
Source: PLoS One. 2019 May 16;14(5):e0216807. doi: 10.1371/journal.pone.0216807 (PMC6522098; doi:10.1371/journal.pone.0216807)
Supplement: S2 Fig — (PDF) [file pone.0216807.s002.pdf]

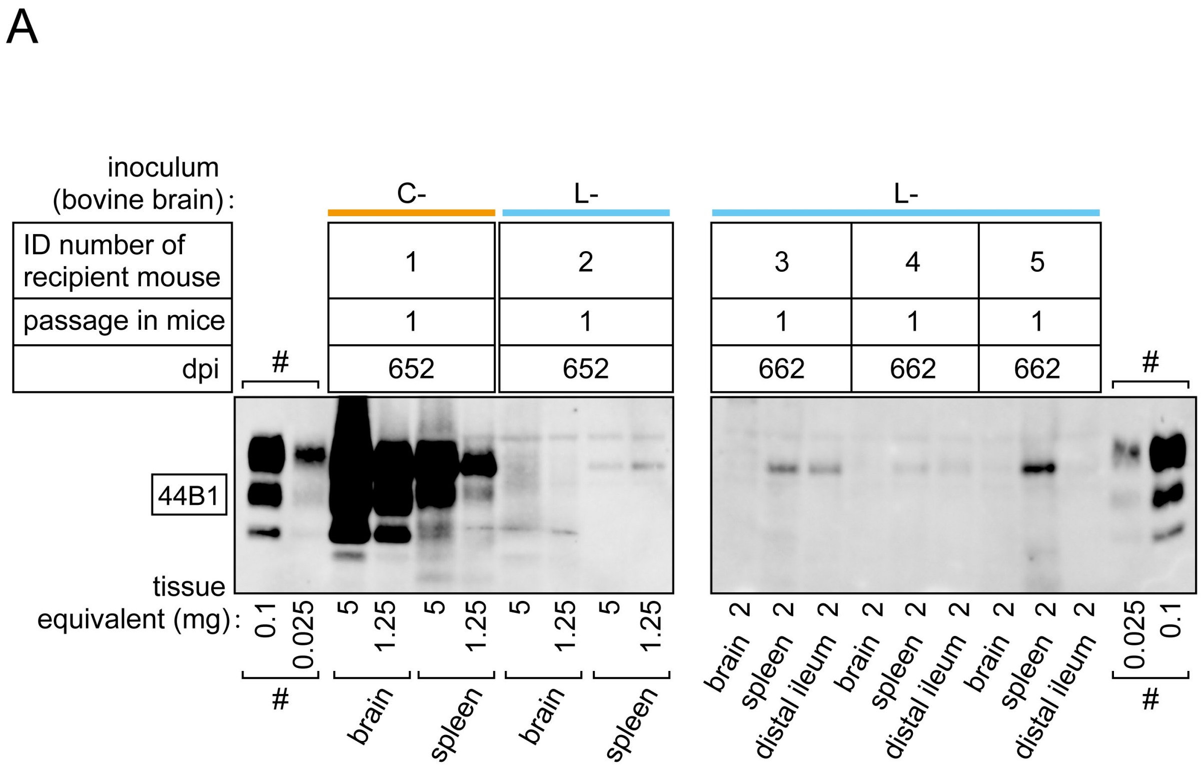
A
inoculum (bovine brain):
C-
L-
L-
ID number of recipient mouse
1
2
3
4
5
passage in mice
1
1
1
1
1
dpi
652
652
662
662
662
#
#
44B1
tissue equivalent (mg):
0.1
0.025
5
1.25
5
1.25
5
1.25
5
1.25
2
2
2
2
2
2
2
2
2
0.025
0.1
#
brain
spleen
brain
spleen
brain
spleen
distal ileum
brain
spleen
distal ileum
brain
spleen
distal ileum
#

B

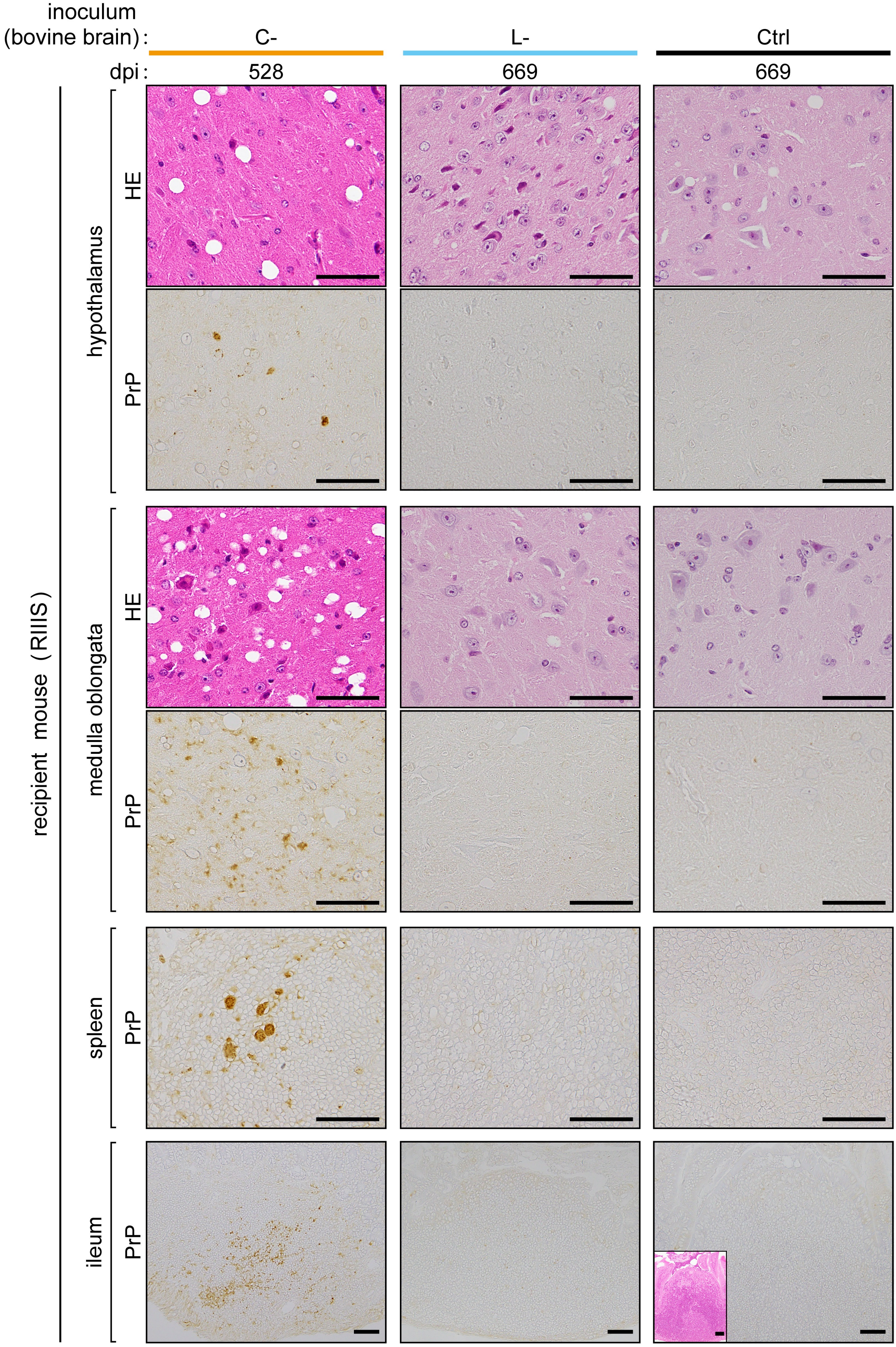

inoculum
(bovine brain) :
C-
L-
Ctrl
dpi :
528
669
669
recipient mouse (RIIIS)
hypothalamus
medulla oblongata
spleen
ileum
HE
PrP
HE
PrP
PrP
PrP

S2 Fig. First challenge of C57BL/6 mice with bovine C- and L-BSE prions.

Western blot analysis of PrP^{Sc} in the brain, spleen, and distal regions of the ileum of representative C57BL/6 mice (ID numbers of 1 to 5) subjected to the first challenge with C- and L-BSE prions from cows. #; thalamus of a C-BSE-affected cow as a positive control. PK digested tissues were subjected to Western blot analysis. PrP^{Sc} was detected using the antibody 44B1. dpi; days post-inoculation. (B) Histopathological and immunohistochemical analysis of representative RIIIS mice challenged with C- and L-BSE prions from cows. Ctrl (control inoculum); saline. HE; hematoxylin-eosin staining. PrP; immunohistochemical staining of PrP^{Sc}. Photographs show hypothalamus, ventral portion of the medulla oblongata, spleen (corresponding to a region of white pulp), and Peyer's patches in distal regions of the ileum (basal lamina are positioned towards the bottom of the photographs as in the inset HE-staining). The scale bars; 50 μm.
